# Comparing population-level mental health of UK workers before and during the COVID-19 pandemic: a longitudinal study using Understanding Society

**DOI:** 10.1136/jech-2021-218561

**Published:** 2022-03-16

**Authors:** Theocharis Kromydas, Michael Green, Peter Craig, Srinivasa Vittal Katikireddi, Alastair H Leyland, Claire L Niedzwiedz, Anna Pearce, Rachel M Thomson, Evangelia Demou

**Affiliations:** 1 MRC/CSO Social and Public Health Sciences Unit, University of Glasgow, Glasgow, UK; 2 Institute of Health and Wellbeing, College of Medical Veterinary and Life Sciences, University of Glasgow, Glasgow, UK

**Keywords:** mental health, COVID-19, occupational health, longitudinal studies, public health

## Abstract

**Objectives:**

The COVID-19 pandemic has substantially affected workers’ mental health. We investigated changes in UK workers’ mental health by industry, socioeconomic class and occupation and differential effects by UK country of residence, gender and age.

**Methods:**

We used representative Understanding Society data from 6474 adults (41 207 observations) in paid employment who participated in pre-pandemic (2017–2020) and at least one COVID-19 survey. The outcome was General Health Questionnaire-12 (GHQ-12) caseness (score: ≥4). Exposures were industry, socioeconomic class and occupation and are examined separately. Mixed-effects logistic regression was used to estimate relative (OR) and absolute (%) increases in distress before and during pandemic. Differential effects were investigated for UK countries of residence (non-England/England), gender (male/female) and age (younger/older) using three-way interaction effects.

**Results:**

GHQ-12 caseness increased in relative terms most for ‘professional, scientific and technical’ (OR: 3.15, 95% CI 2.17 to 4.59) industry in the pandemic versus pre-pandemic period. Absolute risk increased most in ‘hospitality’ (+11.4%). For socioeconomic class, ‘small employers/self-employed’ were most affected in relative and absolute terms (OR: 3.24, 95% CI 2.28 to 4.63; +10.3%). Across occupations, ‘sales and customer service’ (OR: 3.01, 95% CI 1.61 to 5.62; +10.7%) had the greatest increase. Analysis with three-way interactions showed considerable gender differences, while for UK country of residence and age results are mixed.

**Conclusions:**

GHQ-12 caseness increases during the pandemic were concentrated among ‘professional and technical’ and ‘hospitality’ industries and ‘small employers/self-employed’ and ‘sales and customers service’ workers. Female workers often exhibited greater differences in risk by industry and occupation. Policies supporting these industries and groups are needed.

## Introduction

The COVID-19 pandemic has been linked to substantial deteriorations in mental health.^1–3^ Work is a well-established determinant of mental health in working-age people and has been subject to major disruption. Stable and secure jobs are linked to good mental health, while precarious work, income loss and job insecurity have been linked to poorer mental health.^4 5^ Thus far, occupational health research during the pandemic has largely and understandably focused on the needs of healthcare workers.^1 3 6–9^ However, the pandemic also poses a major threat to the mental health of the broader workforce.

In the UK and internationally, employment has been disrupted by high levels of COVID-19 disease and non-pharmaceutical interventions (eg, national/regional ‘lockdown’ measures) introduced to control the pandemic. This disruption, which has varied from country to country, including across the UK,^10^ has impacted on occupational groups in different ways.^1 11–13^ For some, home-working allowed employment to continue but was affected by competing domestic responsibilities (eg, childcare)—often disproportionately affecting women.^2 14 15^ Others have been unable to work but have retained labour market attachment through the UK Government’s ‘furlough’ scheme.^16^ In contrast, more precarious workers (eg, those on ‘zero hours’ contracts) were not well protected by the furlough scheme and, therefore, at risk of job and income loss.

The pandemic has, therefore, been a major shock for employment and work, but its health implications remain underinvestigated. Links between work and mental health are well established^17–20^ and evidence shows the mental health impacts of the pandemic.^2 5 21 22^ We hypothesise that impacts are likely to be differential across industries, social class and occupations, as well as by gender, UK country and infection levels,^23^ due to differences in work-related factors such as job demands, job insecurity and the protective measures implemented at regional or national levels. There is also growing evidence that the economic consequences of the pandemic are particularly negative for young people^24^ and a significant concern for older workers.^11^ We, therefore, investigated how the mental health of UK workers changed during the COVID-19 pandemic by industrial sector, socioeconomic class and occupation, as well as whether any observed changes differed by age, gender and UK country of residence.

## Methods

### Study population

This study uses the UK Household Longitudinal Study (also referred to as ‘Understanding Society’, hereafter abbreviated as ‘Usoc’). Usoc is a nationally representative open-cohort household panel study.^25^ Surveys are normally completed over 24 months, with participants re-interviewed annually. Additional surveys were introduced during the COVID-19 pandemic.^26^ Our sample was restricted to two pre-pandemic surveys (waves 9 and 10/11; data collected: 2017–March 2020) and 6 COVID-19 surveys (April, May, June, July, September and November 2020).^26^ We analysed data from all adults aged 18+ years in wave 9 who were in paid employment (employed, self-employed, both employed and self-employed or in other employment). Participants were included in the analysis if they participated in wave 9 only or in waves 9 and 10/11, and participated in at least one of the COVID-19 surveys.

Proxy respondents, participants ≤18 years old, not in paid employment and with 0 or missing sampling weights were excluded. We also excluded participants with missing data on outcome, exposures and covariates (online supplemental figure S1, table S8). To address this, we reweighted our sample to make it representative of wave 9 participants (online supplemental material: weighting strategy).

10.1136/jech-2021-218561.supp1 Supplementary data

**Figure 1:**
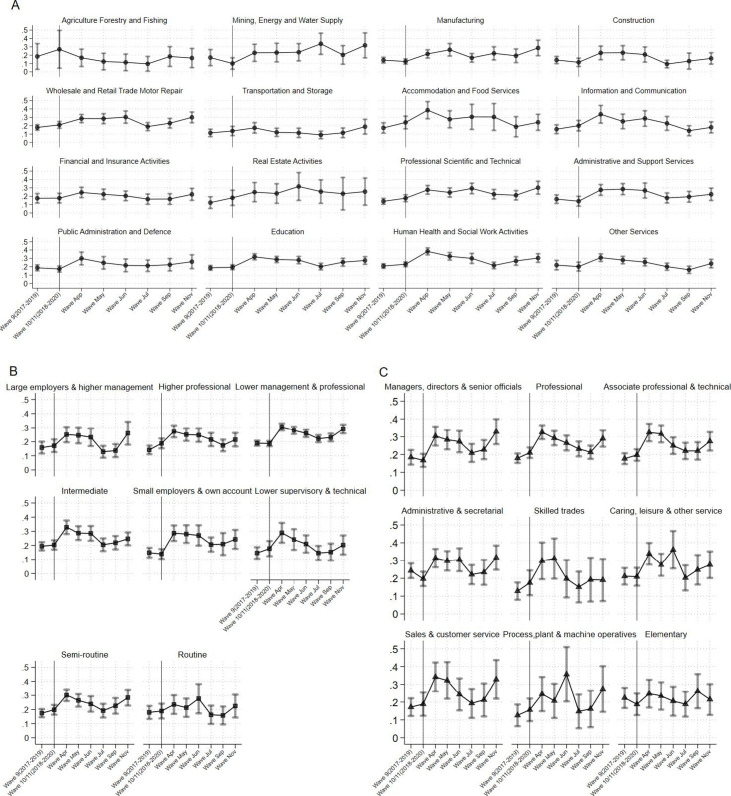
Proportion of GHQ caseness by (A) industry, (B) social class and (C) occupation. GHQ, General Health Questionnaire.

### Outcome

Our outcome of interest was probable psychological distress, defined by ‘caseness’ (probable General Health Questionnaire-12 (GHQ-12) caseness, score ≥4), and was assessed across all included surveys.^27 28^ GHQ-12 is a validated screening tool for psychological distress comprising 12 questions.^29^ Sensitivity analysis considered a lower cut-off of ≥3 (online supplemental figure S4).

### Exposures

Employment-related exposures of interest were industry, socioeconomic class and occupation. Industry was classified using the UK Standard Industrial Classification sections.^30^ Overall, 16 industrial sections were included after adapting the 21 original sections (online supplemental table S1A).^30^ Industry for COVID-19 survey participants was assigned based on the June and July surveys and carried backwards and forwards to all valid baseline participants. Where observations were still missing, data for industrial sector were carried forward first from available wave 10/11 or subsequently from wave 9 data (online supplemental information: transition probabilities).

The National Statistics Socio-Economic Classification (NS-SEC) was used for socioeconomic class categorisation,^31^ carried forward from pre-pandemic to pandemic surveys.

NS-SEC is a theoretically informed, composite measure that only partially captures employment relations and conditions.^32^ Therefore, we also included occupation based on the Standard Occupational Classification 2010, 9 major groups to fully capture occupational effects (online supplemental table S1B).^33^ Occupation was not available in any of the COVID-19 surveys, so we used pre-pandemic data.

We tested the assumption that our exposures remain relatively stable across surveys and over periods spanning one and two waves (pre-pandemic). On average, 92.4%, 88.3% and 87% of participants remained in the same industry, socioeconomic class category and occupation, respectively (online supplemental table S2). Missing information was carried over for around one-fifth of the sample.

### Covariates

We adjusted for potential confounders that might differentially affect change in mental health across employment groups. These were age (continuous variable along with its quadratic term), gender (male/female), UK country of residence (England/non-England (ie, Northern Ireland, Scotland or Wales)), race (white/non-white) and employment type (employed/all other employed (ie, self-employed, both employed and self-employed or in any other type of employment)). Dichotomisation was required due to low numbers.

### Statistical analysis

Sample characteristics were summarised using frequencies and proportions for the pre-pandemic and pandemic periods. We then fitted three mixed-effects generalised logistic regression models with a random intercept for each individual participant to assess odds of GHQ-12 caseness by exposure groupings (industry, socioeconomic class and occupation). All estimations are expressed as ORs and represent the population average for each exposure variable. Predictive margins and average marginal effects in terms of probabilities were estimated to provide an indication of the absolute change in GHQ-12 caseness prevalence in the pandemic period versus pre-pandemic period for each exposure. In all models, the pandemic impact is represented by the interaction between a binary variable for the pre-pandemic period (surveys 9 and 10/11 combined) and pandemic period (April–November surveys) and a categorical variable for industry (Model 1), socioeconomic class (Model 2) and occupation (Model 3). Constituent terms of each interaction are also included in the model. Therefore, the interaction between the variable that distinguishes pre-pandemic and pandemic periods, and our three exposure groupings shows how much greater (OR: >1 in multiplicative terms or ΔP(Y=1)>0 in additive terms) or lower (OR: <1 or ΔP(Y=1)<0) the odds of GHQ-12 caseness associated with the pandemic period was for each exposure category separately compared with pre-pandemicperiod.

We conducted further analysis by estimating three-way interactions between each exposure variable, the binary variable that represents pre-pandemic and pandemic periods and (a) UK country of residence (England/non-England), (b) gender (male/female) and (c) age group (younger: under 50 years or older worker: over 50 years), respectively. These models included the same outcome, exposure and predictor variables and were adjusted for confounding as in Models 1–3 above.

All analysis were conducted in Stata V.17.0.

## Results

Our main unweighted analytical sample (for Models 1–2) comprised 41 207 observations (pre-pandemic: 12 192, pandemic: 29 015) across 6474 individuals (online supplemental table S3: weighted). Most observations (22 738; 55.2%) concern individuals who participated in all 8 waves. Analysis by occupation (Model 3), included 27 110 observations across 4485 individuals (table 1, online supplemental figure S1 and online supplemental tables S4-S6), due to missingness in the occupation variable. Across the main sample, 57.9% were female and mean age was 48.17±11.33 years. Overall, 8.05% were non-white and for most (81.2%) country of residence was England. Most participants (84.5%) were employees.

**Table 1:** Sample characteristics in the pre-pandemic and pandemic Usoc waves* (unweighted sample)

	Full sample (unweighted)					
	Pre-pandemic		Pandemic		Total	
**Outcome**						
GHQ case						
No	10 088		22 070		32 171	
%	82.7		76.1		78.0	
Yes	2104		6945		9055	
%	17.3		23.9		22.1	
**Total**	12 192		29 015		41 207	
**%**	**100.0**		**100.0**		**100.0**	
**Control variables (categorical**)						
Sex						
Male	5235		12 123		17 358	
%	43.0		41.8		42.1	
Female	6957		16 892		23 849	
%	57.1		58.2		57.9	
**Total**	12 192		29 015		41 207	
**%**	**100.0**		**100.0**		**100.0**	
Race						
White	11 124		26 767		37 891	
%	91.2		92.3		92.0	
Non-white	1068		2248		3316	
%	8.8		7.8		8.1	
**Total**	12 192		29 015		41 207	
**%**	**100.0**		**100.0**		**100.0**	
UK country						
England	9874		23 571		33 445	
%	81.0		81.2		81.2	
Wales	721		1684		2405	
%	5.9		5.8		5.8	
Scotland	1098		2662		3760	
%	9.0		9.2		9.1	
Northern Ireland	499		1098		1597	
%	4.1		3.8		3.9	
**Total**	12 192		29 015		41 207	
**%**	**100.0**		**100.0**		**100.0**	
Employment type						
Employed	10 474		24 335		34 809	
%	85.9		83.9		84.5	
Self-employed, both or other	1718		4680		6398	
%	14.1		16.1		15.5	
**Total**	12 192		29 015		41 207	
**%**	**100.0**		**100.0**		**100.0**	
**Control variables (continuous**)						
	**Pre-pandemic**		**Pandemic**		**Total**	
Age	**Mean**	**SD**	**Mean**	**SD**	**Mean**	**SD**
	46.6	11.3	48.8	11.3	48.2	11.3

*Wave 9 response rate for households (ie, at least 1 member responding) was 84.7% and the response rate for individuals (for full interview) was 76.8%;^22^ wave 10/11 response rates are not published as it is a combined survey bridging the gap between the latest annually collected data; and response rates for the COVID-19 surveys for those that were interviewed fully or partially at wave 9 are 42.0% for April, 35.1% for May, 33.5% for June, 32.6% for July, 30.6% for September and 28.6% for November.^20 23–25^

Usoc, Understanding Society.

### Trends in GHQ-12 caseness over time, by industry, socioeconomic class and occupation

Figure 1 shows the weighted prevalence of GHQ-12 caseness by Usoc survey and exposure grouping. Pre-pandemic, GHQ-12 caseness remained relatively stable for all exposures apart for ‘agriculture, forestry and fishing’, ‘mining and quarrying’ and ‘real estate activities’, where the samples are relatively small. Weighted frequencies of all exposures are presented in online supplemental tables S4‒S6. During the pandemic, GHQ-12 caseness increased considerably across all industries at the beginning (April and May; coinciding with the first UK lockdown—announced on 16 March 2020), apart from ‘agriculture, forestry and fishing’ (although with a small sample size), and across all socioeconomic class groups, and occupations.

GHQ-12 caseness subsequently decreased gradually or remained stable until November when the second lockdown in England was announced (31 October 2020). Prevalence of GHQ-12 caseness in this survey rose to levels similar or higher to the first lockdown period. A similar pattern was observed for socioeconomic class; however, no socioeconomic class category showed higher prevalence in November compared with April. Prevalence of GHQ-12 caseness by occupation, resembled that at the start of the first lockdown, with workers in ‘skilled trades’ and ‘elementary’ occupations (eg, labourers, cleaners, services workers, etc), less affected in the second lockdown.

#### Change in GHQ-12 caseness, by industry, socioeconomic class and occupation

Odds of GHQ-12 caseness almost doubled for most industries in the pandemic period (table 2), and trebled for the ‘professional, scientific and technical’ (OR: 3.15, 95% CI 2.17 to 4.57) and ‘manufacturing’ (OR: 3.01, 95% CI 1.92 to 4.74) industries. GHQ-12 caseness by socioeconomic class displayed similar trends, with the likelihood of GHQ-12 caseness in the pandemic period being larger across all categories and ranging from 1.66 (OR: 1.66, 95% CI 1.06 to 2.58) for ‘routine’ class to more than three times (OR: 3.24, 95% CI 2.28 to 4.63) for ‘small employers and own account’. Examining exposure by occupation, GHQ-12 caseness increased across all, with a threefold increase for ‘sales and customer service’ (OR: 3.01, 95% CI 1.61 to 5.62) and a comparable increase for ‘skilled trades occupations’ (OR: 2.88, 95% CI 1.48 to 5.60). The adjusted results demonstrate increased odds, compared with unadjusted, for all exposures. Sensitivity analysis with a lower GHQ-12 caseness cut-off showed few differences mainly due to categories with low precision (online supplemental table S7).

**Table 2:** Unstratified regression models (Models 1–3)

		Interaction multiplicative effect						Interaction additive effect		
		ORs		95% CI†		P values†		Average marginal effect*	95% CI†	P values†
		Unadjusted	Adjusted	Unadjusted	Adjusted	Unadjusted	Adjusted	Adjusted		
Model 1 (Industry SIC 2007)	Accommodation and food services	2.32	2.71	1.57 to 5.40	1.57‒4.68	0.002	0.001	0.11	0.05 to 0.18	0.001
	Agriculture, forestry and fishing	0.56	0.65	0.18 to 1.77	0.2‒2.14	0.323	0.483	−0.04	−0.16 to 0.08	0.528
	Mining, energy and water supply	1.31	1.67	0.58 to 2.98	0.72‒3.86	0.512	0.231	0.06	−0.03 to 0.15	0.210
	Manufacturing	2.40	3.01	1.57 to 3.63	1.92‒4.74	0.001	0.001	0.09	0.05 to 0.13	0.001
	Construction	2.20	2.73	1.29 to 3.77	1.55‒4.81	0.004	0.001	0.08	0.04 to 0.12	0.001
	Wholesale and retail trade motor repair	1.93	2.33	1.37 to 2.71	1.61‒3.38	0.001	0.001	0.08	0.05 to 0.12	0.001
	Transportation and storage	1.40	1.73	0.76 to 2.35	0.95‒3.13	0.243	0.072	0.04	−0.01 to 0.09	0.075
	Information and communication	1.97	2.39	1.18 to 3.30	1.41‒4.07	0.010	0.001	0.08	0.04 to 0.13	0.001
	Financial and insurance activities	1.20	1.42	0.76 to 1.88	0.88‒2.28	0.440	0.148	0.03	−0.01 to 0.07	0.131
	Real estate activities	1.90	2.38	0.77 to 4.73	0.92‒6.14	0.167	0.073	0.09	−0.01 to 0.19	0.070
	Professional, scientific and technical	2.60	3.15	1.85 to 3.67	2.17‒4.57	0.001	0.001	0.10	0.07 to 0.13	0.001
	Administrative and support services	1.79	2.06	1.08 to 2.95	1.22‒3.49	0.024	0.007	0.06	0.02 to 0.10	0.004
	Public administration and defence	1.71	2.20	1.22 to 2.41	1.52‒3.17	0.002	0.001	0.08	0.04 to 0.11	0.001
	Education	2.02	2.44	1.58 to 2.57	1.84‒3.23	0.001	0.001	0.09	0.06 to 0.12	0.001
	Human health and social work activities	1.81	2.18	1.46 to 2.26	1.69‒2.83	0.001	0.001	0.08	0.06 to 0.11	0.001
	Other services	1.87	2.27	1.28 to 2.73	1.52‒3.4	0.001	0.001	0.07	0.04 to 0.11	0.001
(Model 2) Socioeconomic class NS-SEC	Large employers and higher management	1.56	1.91	1.08 to 2.26	1.29‒2.83	0.019	0.001	0.07	0.03 to 0.11	0.001
	Higher professional	2.11	2.60	1.61 to 2.78	1.92‒3.52	0.001	0.001	0.09	0.06 to 0.11	0.001
	Lower management and professional	1.95	2.38	1.66 to 2.29	1.92‒2.95	0.001	0.001	0.08	0.07 to 0.10	0.001
	Intermediate	1.72	2.08	1.33 to 2.23	1.54‒2.8	0.001	0.001	0.07	0.04 to 0.10	0.001
	Small employers and own account	2.60	3.24	1.89 to 3.57	2.28‒4.63	0.001	0.001	0.10	0.07 to 0.14	0.001
	Lower supervisory and technical	1.91	2.31	1.23 to 2.96	1.44‒3.7	0.004	0.001	0.08	0.03 to 0.12	0.001
	Semi-routine	1.79	2.13	1.37 to 2.33	1.57‒2.89	0.001	0.001	0.07	0.04 to 0.10	0.001
	Routine	1.36	1.66	0.9 to 2.06	1.06‒2.58	0.145	0.025	0.05	0.01 to 0.09	0.022
(Model 3) Occupation (SOC)	Managers–directors and senior officials	2.09	2.56	1.52 to 2.86	1.78‒3.67	0.001	0.001	0.10	0.06 to 0.13	0.001
	Professional occupations	1.91	2.34	1.54 to 2.37	1.78‒3.06	0.001	0.001	0.09	0.06 to 0.12	0.001
	Associate professional and technical occupations	2.00	2.47	1.52 to 2.64	1.78‒3.42	0.001	0.001	0.09	0.06 to 0.12	0.001
	Administrative and secretarial occupations	1.55	1.89	1.14 to 2.09	1.32‒2.7	0.00	0.001	0.07	0.03 to 0.11	0.001
	Skilled trades occupations	2.32	2.88	1.25 to 4.32	1.48‒5.6	0.008	0.002	0.09	0.04 to 0.15	0.002
	Caring–leisure and other service occupations	1.82	2.17	1.28 to 2.59	1.45‒3.24	0.001	0.00	0.07	0.03 to 0.11	0.001
	Sales and customer service occupations	2.50	3.01	1.38 to 4.54	1.61‒5.62	0.003	0.001	0.11	0.05 to 0.17	0.001
	Process–plant and machine operatives	2.01	2.47	1.08 to 3.75	1.28‒4.74	0.029	0.007	0.07	0.02 to 0.13	0.011
	Elementary occupations	1.26	1.60	0.8 to 1.99	0.98‒2.61	0.320	0.063	0.05	−0.01 to 0.10	0.058

Note: grey-shadowed areas represent estimations for the unadjusted models.

*Show the change in probability of GHQ caseness from pre-pandemic period to pandemic period.

†CIs and p values are estimated for the interaction term separately for each value of our exposure variables.

GHQ, General Health Questionnaire; NS-SEC, National Statistics Socio-Economic Classification; SIC, Standard Industrial Classification.

**Figure 2:**
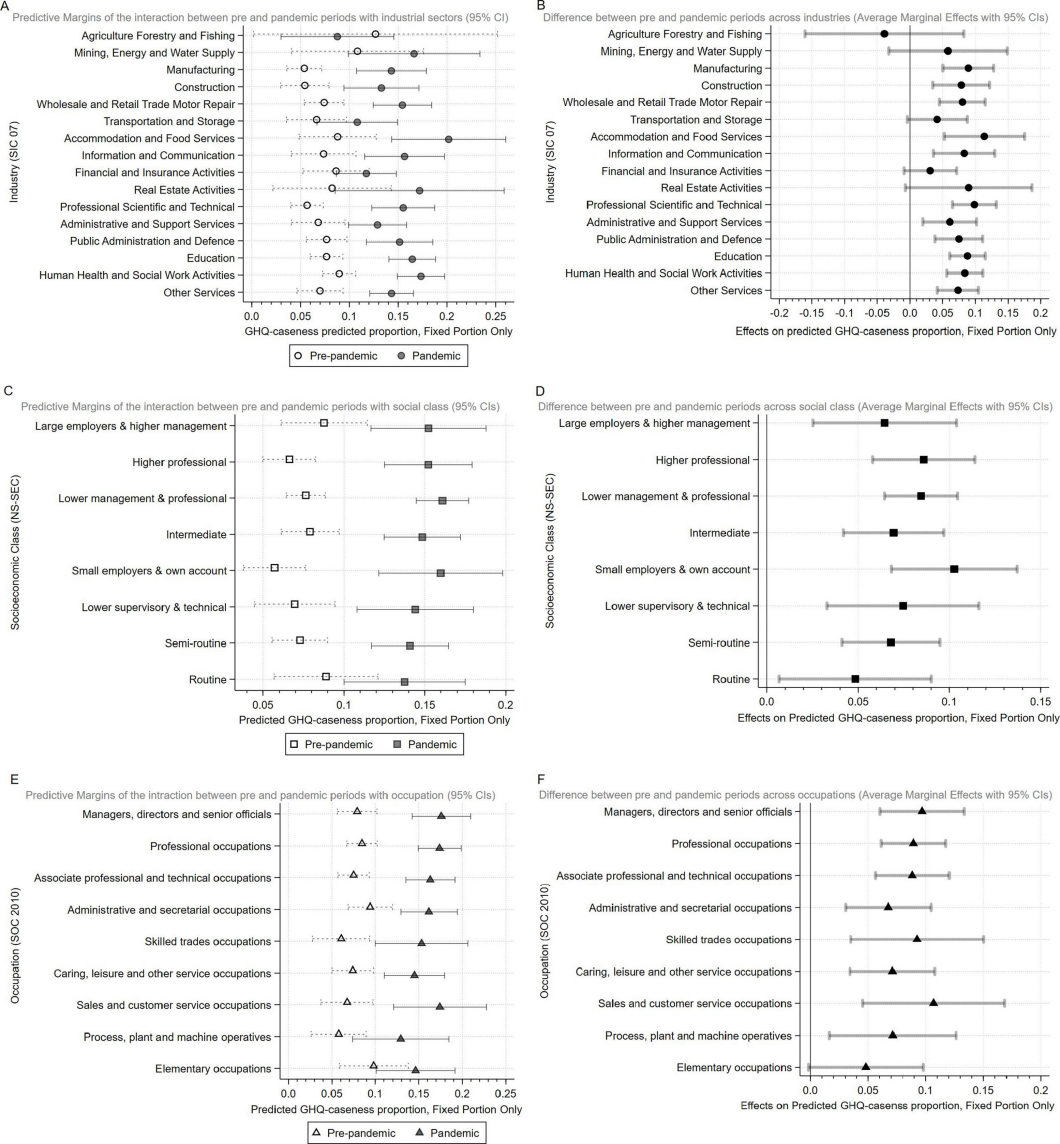
Predictive margins and average marginal effects for all exposures for pre-pandemic and pandemic periods (A, C and E) and the change in probability between pre- and pandemic period (B, D and F).

Figure 2 shows predicted absolute changes in mental health over the pandemic period. While the direction of the results mainly remained the same, the industries hardest hit varied. The ‘professional, scientific and technical’ and ‘manufacturing’ industries experienced the biggest relative increase in GHQ-12 caseness, but ‘accommodation and food services’ experienced the greatest absolute increase (11%). All socioeconomic class groups saw absolute increases in GHQ-12 caseness, and in line with the ORs results ‘small employers and own account’ saw the biggest absolute increase (figure 2C). For occupation, ‘sales and customer service’, ‘skilled trades’ and ‘associate professional and technical’ occupations saw large absolute increases in GHQ-12 caseness; however, change among ‘managers–directors and senior officials’ and ‘professional’ occupations was comparable.

#### Gender, age and UK country of residence differences

Analyses where UK country of residence was part of the three-way interaction demonstrated little evidence of differences. Similar findings were observed for age. Exceptions were found in the ‘public administration and defence‘ and ‘construction’ industries, where non-England residents and younger workers, respectively, displayed higher odds of GHQ-12 caseness compared with their counterparts, respectively. By socioeconomic class, older ‘routine’ and younger ‘semi-routine’ workers fared worse, whereas by occupation non-England workers in ‘elementary’ occupations, and younger ‘professionals’ (online supplemental figures S2,S3) displayed higher odds in GHQ-12 caseness compared with their counterparts.

**Figure 3:**
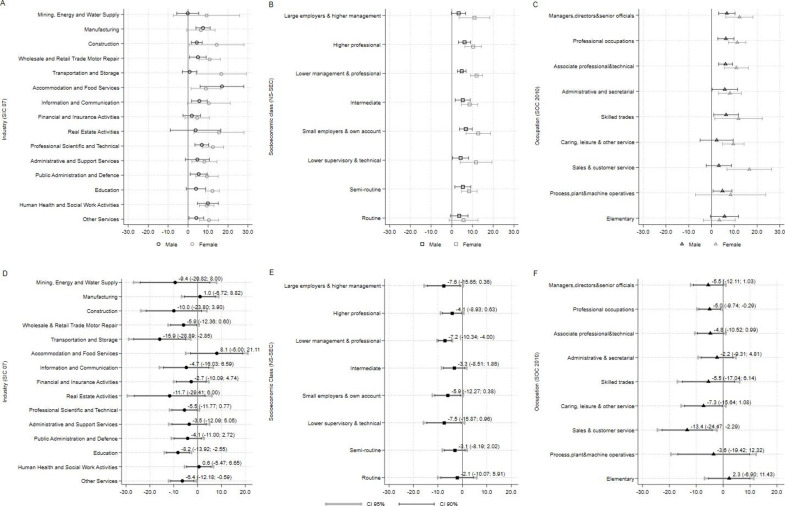
Average Marginal Effects (AVE) by gender for all exposures (A,B, C) and their percentage point probability difference (Δ(AVE) (D,E,F)

Regarding gender, differences in GHQ-12 caseness were apparent in several industries (figure 3). The effects were consistently larger for women in ‘transportation and storage’ (Δ(AVE): −15.9pp; 95% CI: −28.89 to −2.85), ‘education’ (Δ(AVE): −8.2pp; 95% CI: −13.36 to −2.55) and ‘other services’ (Δ(AVE): −6.4; 95% CI: −12.18 to −0.59). Other industries showed similarly larger effects for women but at a lower CI level (figure 3). By socioeconomic class, mental health deteriorated across most groups for both genders, but in ‘lower management and professional’ (Δ(AVE): −7.2pp; 95% CI: −10.34 to −4.00) women were affected more. Similar results were observed in other socioeconomic class groups, although at lower CI levels (figure 3). By occupation, women in ‘sales and customer service’ exhibited the largest difference (Δ(AVE): −13.4pp; 95% CI: −24.47 to −2.29) followed by ‘professional occupations’ (Δ(AVE): −5.0pp; 95% CI: −9.74 to −0.29). Similar to industry and socioeconomic class, differences were also observed in other occupations at a lower CI level (figure 3).

## Discussion

### Summary of findings

Psychological distress increased substantially in UK workers during the COVID-19 pandemic across almost all industries, socioeconomic class and occupational groups. In most industries, odds of GHQ-12 caseness increased more than twofold, with workers in the ‘professional, scientific and technical’, ‘hospitality’, ‘construction’ and ‘manufacturing’ industries being most impacted. By socioeconomic class, small employers and the self-employed were most adversely affected, with odds increasing more than three times, while for most of the other socioeconomic class groups the odds of GHQ-12 caseness increased more than twofold. During the pandemic, occupations in ‘sales and customer service’ and ‘skilled trades’ were most affected. Analysis using three-way interactions showed little evidence of significant differences in odds of GHQ-12 caseness by UK country of residence and age, with a few exceptions. Marked gender differences with women affected more, and were observed across all exposures and particularly in the ‘transportation and storage’ and ‘education’ industries, the ‘lower supervisory and technical’ socio-economic class group and in ‘sales and customer service’ occupations.

### Findings in context with previous literature

Our findings are in line with national statistics demonstrating increasing rates of work-related mental health issues, particularly in public service industries such as education; health and social care; and public administration and defence.^34^ While our findings support this, we also show the impact of the pandemic on other industries. Our findings demonstrated an increase in GHQ-12 caseness across most occupations; however, workers in some occupations with high levels of unstable working conditions, low autonomy and job control, technical tasks and female workers in occupations such as sales and customer service representatives and professionals were most adversely affected.

Most research to date during the pandemic has focused on the health and well-being of healthcare workers.^35–38^ Other frontline workers, working in areas with higher rates of contagion also report higher levels of stress and burnout and lower levels of compassion satisfaction.^35^ Our results support the findings that frontline healthcare workers require further support, and that targeted prevention and intervention programmes are necessary. However, we also demonstrate that other industries and occupational groups are at risk and require specific attention. These may not have the stability or safety nets in place and/or could not continue with their occupations by working from home, including those in manufacturing, construction, hospitality, the self-employed and small business owners, and occupations that involve unstable working conditions or the performance of technical tasks.

### Study strengths and limitations

Our study has several strengths. We used a large nationally representative longitudinal dataset to examine differences in GHQ-12 caseness during the COVID-19 pandemic across industries, socioeconomic class groups and occupations, which fills an important gap in the literature. Our analysis included pre-pandemic outcome measures and six surveys of data collection after the start of the pandemic, allowing us to examine trends before and after the initial lockdown. We also explored multiple dimensions of employment and heterogeneity across groups. Some limitations should be noted. First, while estimates were weighted to adjust for survey, non-participation residual bias cannot be excluded. Second, there were changes in the modality of administration of the COVID-19 surveys compared with pre-pandemic surveys (from mixed mode to online), which may have affected the findings. However, empirical investigation suggests this is unlikely to have biased responses.^39^ The pandemic context may have also influenced participant reporting more broadly. Furthermore, observations in some industries (agriculture, forestry and fishing: N=342; mining, energy and water supply: N=744 and real estate activities: N=485) and by country of residence and age were relatively limited, requiring broad classifications and/or reducing the precision of estimates. The three-way interaction analyses displayed relatively small differences and wide CIs and we suggest these analyses be replicated when more COVID-19 surveys are available. Discrepancies between socioeconomic class and occupational exposure groups that may cover the same jobs (eg, ‘routine’ socioeconomic class and ‘elementary’ occupations) highlight the need to use more detailed occupational breakdowns. We only included one mental health measure and results may differ using other measures of depression or anxiety. Further research examining clinical outcomes (eg, receipt of antidepressant prescription) is also needed.

### Policy implications and conclusions

Our study has important implications for public and occupational health policy. The increase in GHQ-12 caseness seen in UK workers highlights the trade-off between protecting the public’s physical health during the pandemic and the adverse effects this could have. Our findings of substantial disparities in GHQ-12 caseness across industries, socioeconomic class groups, occupations, as well as some differences between genders and regions, highlight the complexity of the extended health harms associated with the pandemic and risks to mental health. Finding that particular groups such as workers in the ‘professional, scientific and technical’, ‘hospitality’, ‘construction’ and ‘manufacturing’ industries, small employers and the self-employed and particularly female workers have been disproportionately affected illustrates broader inequalities in the job market and employment conditions.^12 13 24 40^ The differences by UK country of residence may reflect pre-existing labour markets, as well as decentralised handling of the lockdown measures. The self-employed and small employers may have fewer safety nets and greater fear of the lack of employment opportunities after the pandemic. Further research is needed to understand the risk factors and mechanisms that are driving these findings for each group and whether the substantial increase in GHQ-12 caseness remains as lockdown measures are eased and working environments establish their new ‘normal’. The deterioration of mental health is also expected to create additional capacity problems for healthcare services, given the strain these services are already under. Monitoring the mental health of the working-age population can inform workplace and non-workplace support measures that are needed. Employers need to consider tailored workplace practices for mental health problems, including enhanced psychological support and measures to protect jobs and incomes.^41^ Furthermore, policies tailored to these exposures are needed prioritising for instance support to the self-employed/small business owners, and particular demographic groups (eg, women in ‘sales and customer service’ occupations, younger ‘construction’ or non-England workers in ‘public administration and defence’) with high risk.

**What is already known on this topic**

- Employment has been disrupted by the COVID-19 pandemic and non-pharmaceutical interventions (eg, national and regional ‘lockdowns’) introduced to control the pandemic. The pandemic has impacted on different occupational groups in different ways and has been linked to substantial deteriorations in mental health.

**What this study adds**

- The effect of the COVID-19 pandemic on mental health has been particularly pronounced for those working in professional and technical industries, hospitality, customer service occupations, small employers and the self-employed as well as female workers.

**How this study might affect research, practice or policy**

- Monitoring the mental health of the working-age population can inform workplace and non-workplace support measures that are needed. Policies should prioritise support to certain industries, occupations, the self-employed/small business owners, and particular demographic groups (eg, women in sales and customer service occupations, younger ‘construction’ or non-England workers in ‘Ppublic Aadministration and Ddefence’) with high risk.

## Data Availability

Data are available in a public, open access repository. Understanding Society (Usoc) data are available through the UK Data Service. Researchers who would like to use Usoc need to register with the UK Data Service before being allowed to apply for or download datasets (https://www.understandingsociety.ac.uk/documentation/access-data).
