# Supplementary material for: Comparing population-level mental health of UK workers before and during the COVID-19 pandemic: a longitudinal study using Understanding Society
Source: J Epidemiol Community Health. 2022 Mar 16:jech-2021-218561. doi: 10.1136/jech-2021-218561 (PMC8931794; doi:10.1136/jech-2021-218561)
Supplement: Supplementary file 1 [file jech-2021-218561supp001.pdf]

Comparing population-level mental health of UK workers before and during the COVID-19 pandemic: A longitudinal study using Understanding Society

Supplementary Material

Table S1A. SIC Industrial Classifications Sections in Understanding Society and study

**SIC Industrial Classifications Sections - Understanding Society**		**SIC Industrial Classifications Sections -Included in Study**
Agriculture, Forestry and Fishing	1	Agriculture, Forestry and Fishing
Mining and Quarrying	2	Mining, Energy and Water Supply
Electricity, Gas, Steam and Air Conditioning		
Water Supply; Sewerage, Waste Management and Remediation Activities		
Manufacturing	3	Manufacturing
Construction	4	Construction
Wholesale and Retail Trade	5	Wholesale and Retail Trade Motor Repair
Repair of Motor Vehicles and Motorcycles		
Transportation and Storage	6	Transportation and Storage
Accommodation and Food Service Activities	7	Accommodation and Food Services
Information and Communication	8	Information and Communication
Financial and Insurance Activities	9	Financial and Insurance Activities
Real Estate Activities	10	Real Estate Activities
Professional, Scientific and Technical Activities	11	Professional Scientific and Technical
Administrative and Support Service Activities	12	Administrative and Support Services
Public Administration and Defence; Compulsory Social Security	13	Public Administration and Defence
Education	14	Education
Human Health and Social Work Activities	15	Human Health and Social Work Activities
Arts, Entertainment and Recreation	16	Other Services
Other Service Activities		
Activities of Households as Employers		

Table S1B. Social class by occupation

Socio-economic class (NS-SEC) /Occupation (SOC 2010)	Managers, directors and senior officials	Professional occupations	Associate professional and technical occupations	Administrative and secretarial occupations	Skilled trades occupations	Caring, leisure and other service occupations	Sales and customer service occupations	Process, plant and machine operatives	Elementary occupations	Total
Large employers & higher management	1,026	371	280	26	14	0	15	6	2	1,740
%	30.81	5.43	5.32	0.74	1.03	0	0.97	0.53	0.12	6.42
Higher professional	60	2,444	226	28	14	11	10	7	0	2,800
%	1.80	35.79	4.29	0.79	1.03	0.45	0.65	0.61	0	10.33
Lower management & professional	1,799	3,498	3,722	795	146	117	203	34	47	10,361
%	54.02	51.22	70.69	22.48	10.75	4.79	13.11	2.98	2.84	38.22
Intermediate	59	271	601	2,030	90	395	362	11	21	3,840
%	1.77	3.97	11.42	57.39	6.63	16.16	23.37	0.96	1.27	14.16
Small employers & own account	309	195	160	187	393	315	80	152	177	1,968
%	9.28	2.86	3.04	5.29	28.94	12.89	5.16	13.31	10.69	7.26
Lower supervisory & technical	46	5	110	58	426	173	113	274	169	1,374
%	1.38	0.07	2.09	1.64	31.37	7.08	7.3	23.99	10.21	5.07
Semi-routine	30	39	146	381	184	1,334	748	243	509	3,614
%	0.90	0.57	2.77	10.77	13.55	54.58	48.29	21.28	30.74	13.33
Routine	1	6	20	32	91	99	18	415	731	1,413
	0.03	0.09	0.38	0.9	6.7	4.05	1.16	36.34	44.14	5.21
Total	**3,330**	**6,829**	**5,265**	**3,537**	**1,358**	**2,444**	**1,549**	**1,142**	**1,656**	**27,110**
	**100**	**100**	**100**	**100**	**100**	**100**	**100**	**100**	**100**	**100**

Weighting strategy

First, we created an inverse-probability weight within each survey wave for having outcome data based on:

i. Age, split in three broad age bands (1: 18/29y old, 2: 30/45y old and 3: Over 45y old),
ii. gender (male vs female),
iii. race (white vs non-white) and
iv. type of employment which is another binary variable that categorises employees versus those that are either self-employed, both employees and self-employed and other.

These were combined with weights provided with the COVID-19 surveys, which were designed to weight the observed sample at each COVID survey to resemble the sample from the 2017-2019 survey. We also weighted the 2018-2020 survey to resemble the 2017-2019 survey based on the same covariates listed above plus the binary variable for psychological distress and combined this with the weight for outcome missingness for that survey. By using this method, each individual has a non-monotone weight at each survey wave.

Transition Probabilities

A detailed description of the method we have used to carried observations forward and backwards for the industry variables can be found below:

1. We first recoded the industry variable in the Covid-19 surveys where data was available (June and July to align with the industry variable we have used for the pre-pandemic Waves
2. Then we carried forward valid observations (not missing or inapplicable) for September Wave from July first and if still missing from June only to those that were employed in both waves and valid observations for November using the exact same method.
3. Then we carried backwards observed data from June and July for April and May.
4. Then, we have carried forward or backwards information for June and July where we only had observed information for the same individual in just one of the two surveys.
5. Where data for June and July was still missing, we have carried forward information from pre-pandemic (Wave 9 and/or Wave 10/11). Steps 4 and 5 have resulted into enhancing our sample by 115 observations in June and 77 observations in July.
6. Then we carried pre-pandemic information forward for all COVID-19 surveys where data was still missing.

Using these steps and before we apply any restrictions to the data for the purpose of our analysis apart from being employed, and after we have employed our data carrying forward and backward

method we had 73,825 valid observations across 20,886 individuals for both pre-pandemic Waves 9 and 10/11 and all Covid-19 surveys (Pre-pandemic: 31,739 observations, Pandemic: 42,086 observations) of which 15,943 observations were observed during pandemic while all data points for pre-pandemic were observed. Therefore, we have carried forward or backwards 26,143 observations for all six COVID-19 surveys (June and July included) of which 5,455 (20.8%) observations were carried forward from pre-pandemic waves (Waves 9 and Wave 10/11).

We then imposed our exclusion criteria in the data and come up with an analytical sample of 41,207 observations, where we ended up with 41,207 observations (Pre-Pandemic: 12,192, Pandemic: 29,015). Of them 9,781 observations are observed data points that refer to individuals that participated in both pre and pandemic periods (but after COVID-19 June survey where data for industry was first observed) and 19,234 observations were carried either forward or backwards from pre or pandemic Waves.

We have used the same strategy to carry information forwards for the other two exposures variables we have used (social class and occupation).

Table S2.: Transition probabilities and correlations between current and lagged values for industry, social class, and occupation

Industry	Pre pandemic		Pandemic	Social class	Pre pandemic		Occupation	Pre pandemic	
	2-wave	1-wave	1-month PP		2-wave	1-wave		2-wave	1-wave
Agriculture Forestry and Fishing	93.74	94.02	90.32	Large employers & higher management	85.59	85.64	Managers, directors and senior officials	87.29	87.57
Mining, Energy and Water Supply	91.91	91.79	79.51	Higher professional	92.83	92.72	Professional occupations	93.20	93.05
Manufacturing	93.61	93.41	87.39	Lower management & professional	91.85	91.79	Associate professional and technical occupations	86.19	86.29
Construction	93.20	93.55	89.69	Intermediate	87.38	87.26	Administrative and secretarial occupations	86.04	85.74
Wholesale and Retail Trade Motor Repair	91.23	91.15	89.51	Small employers & own account	92.23	92.24	Skilled trades occupations	87.73	87.80
Transportation and Storage	93.95	93.94	79.52	Lower supervisory & technical	83.40	83.44	Caring, leisure and other service occupations	89.58	89.66
Accommodation and Food Services	84.48	84.41	74.12	Semi-routine	87.22	87.19	Sales and customer service occupations	80.22	80.87
Information and Communication	92.66	92.64	79.30	Routine	85.65	85.79	Process, plant and machine operatives	88.95	88.84
Financial and Insurance Activities	93.78	93.68	90.22				Elementary occupations	81.94	82.66
Real Estate Activities	92.69	92.67	85.51						
Professional Scientific and Technical	92.90	92.88	72.31						
Administrative and Support Services	87.26	87.27	54.38						
Public Administration and Defence	94.58	94.79	77.32						
Education	96.00	95.87	96.41						
Human Health and Social Work Activities	96.54	96.48	89.47						
Other Services	90.30	90.14	62.96						
**Average**	**92.43**	**92.42**	**81.12**		**88.27**	**88.26**		**86.79**	**86.94**

Table S3: Sample characteristics in the pre and pandemic Waves (Weighted Sample)

	Full sample (Weighted)		
	Pre-Pandemic	Pandemic	Total
Outcome			
GHQ case %			
No	82.06	75.34	77.34
Yes	17.94	24.66	22.66
Total	100	100	100
Control Variables (Categorical)			
Sex %			
Male	46.68	49.60	48.73
Female	53.32	50.22	51.27
Total	100	100	100
Race %			
White	93.77	93.73	93.74
Non-White	6.23	6.27	6.26
Total	100	100	100
UK country %			
England	86.60	85.21	85.63
Wales	3.94	4.32	4.21
Scotland	7.81	8.06	7.98
Northern Ireland	1.64	2.41	2.18
Total	100	100	100
Employment Type %			
Employed	86.42	85.02	85.41
Self-Employed, both or other	13.58	14.98	14.56
Total	100	100	100

Control Variables (Continuous)

	Pre-pandemic		Pandemic		Total	
Age	mean	sd	mean	sd	mean	sd
	45.20	11.86	45.92	12.21	45.70	12.11

Table S4: Observations for Industry (SIC-2007) in pre-pandemic and pandemic Waves (Unweighted and Weighted Sample (in brackets))

Industry SIC-2007	Full sample		
	Pre-pandemic	Pandemic	Total
Agriculture Forestry and Fishing	58	284	342
%	0.47 (0.45)	0.98 (1.22)	0.83 (0.99)
Mining, Energy and Water Supply	171	573	744
%	1.4 (1.31)	1.97 (1.95)	1.8 (1.76)
Manufacturing	1,038	1,877	2,915
%	8.51 (8.54)	6.47 (7.01)	7.07 (7.47)
Construction	533	1,311	1,844
%	4.37 (4.79)	4.52 (5.90)	4.47 (5.57)
Wholesale and Retail Trade Motor Repair	1,326	2,748	4,074
%	10.88 (11.86)	9.47 (11.94)	9.89 (11.91)
Transportation and Storage	492	962	1,454
%	4.04 (4.18)	3.32 (4.19)	3.53 (4.19)
Accommodation and Food Services	344	670	1,014
%	2.82 (2.93)	2.31 (2.72)	2.46 (2.78)
Information and Communication	495	1,314	1,809
%	4.06 (4.21)	4.53 (4.44)	4.39 (4.37)
Financial and Insurance Activities	430	1 354	1,784
%	3.52 (3.86)	4.67 (4.25)	4.33 (4.13)
Real Estate Activities	159	326	485
%	1.30 (1.32)	1.12 (0.90)	1.18 (1.03)
Professional Scientific and Technical	948	1 853	2,801
%	7.78 (7.84)	6.39 (5.37)	6.80 (6.11)
Administrative and Support Services	465	1,292	1,757
%	3.81 (4.02)	4.45 (3.72)	4.26 (3.81)
Public Administration and Defence	1,054	1,465	2,519
%	8.65 (7.89)	5.05 (4.51)	6.11 (5.52)
Education	1,820	4,653	6,473
%	14.93 (14.41)	16.04 (13.59)	15.71 (13.83)
Human Health and Social Work Activities	2,266	4,360	6,626
%	18.59 (17.41)	15.03 (13.63)	16.08 (14.76)
Other Services	593	3,973	4,566
%	4.86 (4.97)	13.69 (14.66)	11.08 (11.77)
Total	12,192	29,015	41,207
%	100	100	100

Table S5:Observations for Social Class (NS-SEC) pre-pandemic and pandemic Waves (Unweighted and Weighted Sample (in brackets))

	Full sample		
Social Class NS-SEC	**Pre-pandemic**	**Pandemic**	**Total**
Large employers & higher management	715	1,686	2,401
%	5.86 (5.92)	5.81 (5.32)	5.83 (5.50)
Higher professionals	1,351	3,214	4,565
%	11.08 (10.41)	11.08 (9.15)	11.08 (9.52)
Lower management & pr	4,370	10,455	14,825
%	35.84 (35.17)	36.03 (31.28)	35.98 (32.44)
Intermediate	1,697	4,164	5,861
%	13.92 (13.99)	14.35 (13.98)	14.22 (13.98)
Small employers & own account	1,108	2,471	3,579
%	9.09 (8.90)	8.52 (8.47)	8.69 (8.60)
Lower supervisory & technical	700	1,612	2,312
%	5.74 (6.17)	5.56 (7.48)	5.61 (7.09)
Semi-routine	1,622	3,888	5,510
%	13.30 (13.88)	13.4 (16.45)	13.37 (15.68)
Routine	629	1,525	2,154
%	5.16 (5.56)	5.26 (7.88)	5.23 (7.19)
Total	**12,192**	**29,015**	**41,207**
%	**100**	**100**	**100**

BMJ Publishing Group Limited (BMJ) disclaims all liability and responsibility arising from any reliance placed on this supplemental material which has been supplied by the author(s)

Table S6:Observations for Occupations (SoC) in pre-pandemic and pandemic Waves (Unweighted and Weighted Sample (in brackets))

	Full sample		
Occupations SOC 2010, condensed	**Pre-pandemic**	**Pandemic**	**Total**
Managers, directors and senior official	978	2,352	3,330
%	11.91 (12.17)	12.44 (12.01)	12.28 (12.06)
Professional occupations	2,051	4,778	6,829
%	24.97 (23.29)	25.28 (20.12)	25.19 (21.07)
Associate professional and technical occupations	1,565	3,700	5,265
%	19.06 (18.76)	19.58 (18.53)	19.42 (18.60)
Administrative and secretarial occupations	1,055	2,482	3,537
%	12.85 (12.51)	13.13 (10.51)	13.05 (11.11)
Skilled trades occupations	420	938	1,358
%	5.12 (5.72)	4.96 (7.89)	5.01 (7.24)
Caring leisure and other service occupations	775	1,669	2,444
%	9.44 (9.20)	8.83 (9.62)	9.02 (9.49)
Sales and customer service occupations	500	1,049	1,549
%	6.09 (6.67)	5.55 (7.26)	5.71 (7.09)
Process, plant, and machine operatives	358	784	1,142
%	4.36 (4.47)	4.15 (5.31)	4.21 (5.06)
Elementary occupations	508	1,148	1,656
%	6.19 (7.20)	6.07 (8.75)	6.11 (8.28)
**Total**	**8,210**	**18,900**	**27,110**
**%**	**100**	**100**	**100**

Table S7. Unstratified Regression models – Sensitivity analysis for outcome variable ((0: 0≤GHQ-12≤2, 1: 3≤GHQ-12≤12).

		Odds Ratios		P values		CI 95%	
		Analytical Model	Sensitivity	Analytical Model	Sensitivity	Analytical Model	Sensitivity
Industry SIC 2007 (Model 1)	Accomm&Food Services	2.710	2.372	0.000	0.001	1.569–4.680	1.435–3.939
	Agriculture Forestry and Fishing	0.655	0.525	0.483	0.301	0.201–2.136	0.155–1.784
	Mining–Energy and Water Supply	1.670	1.841	0.231	0.136	0.722–3.863	0.827–4.117
	Manufacturing	3.013	3.043	0.000	0.000	1.917–4.737	2.051–4.531
	Construction	2.726	2.153	0.001	0.016	1.545–4.807	1.156–4.031
	Wholesale and Retail Trade Motor Repair	2.333	2.358	0.000	0.000	1.611–3.380	1.651–3.373
	Transportation and Storage	1.725	2.014	0.072	0.014	0.952–3.126	1.118–3.37
	Information and Communication	2.393	2.107	0.001	0.001	1.406–4.073	1.34–3.356
	Financial and Insurance Activities	1.420	1.531	0.148	0.065	0.883–2.282	0.977–2.413
	Real Estate Activities	2.378	2.502	0.073	0.063	0.922–6.135	0.954–6.596
	Professional Scientific and Technical	3.150	3.461	0.000	0.000	2.169–4.575	2.454–4.908
	Administrative and Support Services	2.061	1.852	0.007	0.019	1.218–3.488	1.105–3.097
	Public Administration and Defence	2.197	2.356	0.000	0.000	1.522–3.172	1.691–3.287
	Education	2.435	2.689	0.000	0.000	1.835–3.232	2.047–3.532
	Human Health and Social Work Activities	2.185	2.266	0.000	0.000	1.687–2.830	1.788–2.876
	Other Services	2.273	2.155	0.000	0.000	1.517–3.404	1.494–3.121
Social class NS-SEC (Model 2)	Large employers & higher management)	1.907	1.893	0.001	0.001	1.287–2.827	1.324–2.75
	Higher professional	2.601	2.810	0.000	0.000	1.919–3.524	2.111–3.694
	Lower management & professional	2.381	2.477	0.000	0.000	1.924–2.947	2.028–3.02
	Intermediate	2.078	2.159	0.000	0.000	1.544–2.796	1.622–2.873
	Small employers & own account	3.244	3.180	0.000	0.000	2.276–4.626	2.217–4.582
	Lower supervisory & technical	2.308	2.160	0.000	0.001	1.441–3.697	1.402–3.345
	Semi-routine	2.132	2.191	0.000	0.000	1.575–2.887	1.649–2.914
	Routine	1.656	1.649	0.025	0.021	1.065–2.577	1.08–2.531
Occupation (SOC) (Model 3)	Managers, directors and senior officials	2.557	2.369	0.000	0.000	1.783–3.667	1.704–3.279
	Professional occupations	2.337	2.621	0.000	0.000	1.783–3.064	2.037–3.386
	Associate professional and technical occupations	2.471	2.664	0.000	0.000	1.784–3.424	1.954–3.622
	Administrative and secretarial occupations	1.892	1.925	0.000	0.000	1.324–2.703	1.382–2.688
	Skilled trades occupations	2.877	3.068	0.002	0.001	1.479–5.597	1.571–6.007
	Caring, leisure and other service occupations	2.166	2.364	0.000	0.000	1.448–3.239	1.624–3.452
	Sales and customer service occupations	3.010	3.064	0.001	0.000	1.613–5.618	1.643–5.647
	Process, plant and machine operatives	2.467	2.457	0.007	0.003	1.283–4.742	1.361–4.459
	Elementary occupations	1.596	1.471	0.063	0.101	0.976–2.612	0.928–2.337

Note: Grey-shadowed areas represent estimations for the sensitivity analysis

Table S8. Total, included and excluded observations and participants across all waves used

Waves	Observations			Individuals		
	Total	Included	Excluded	Total	Included	Excluded
Pre-Pandemic						
Wave 9	36,055	6,450	29,605	14,884	1,162	13,722
Wave 10/11	33,382	5,742	27,640	11,911	2,149	9,762
COVID-19 April Survey	17,761	5,679	12,082	3,367	951	2,416
COVID-19 May Survey	15,529	5,153	10,376	2,491	778	1,713
COVID-19 June Survey	14,123	4,971	9,152	2,066	729	1,337
COVID-19 July Survey	13,574	4,810	8,764	2,014	703	1,311
COVID-19 September Survey	12,876	4,326	8,550	1,774	599	1,175
COVID-19 November Survey	12,846	4,076	8,770	1,738	565	1,173
Total	**155,906**	**41,207**	**114,699**	**40,245**	**6,474**	**33,771**

Figure S1: Flowchart of study participants

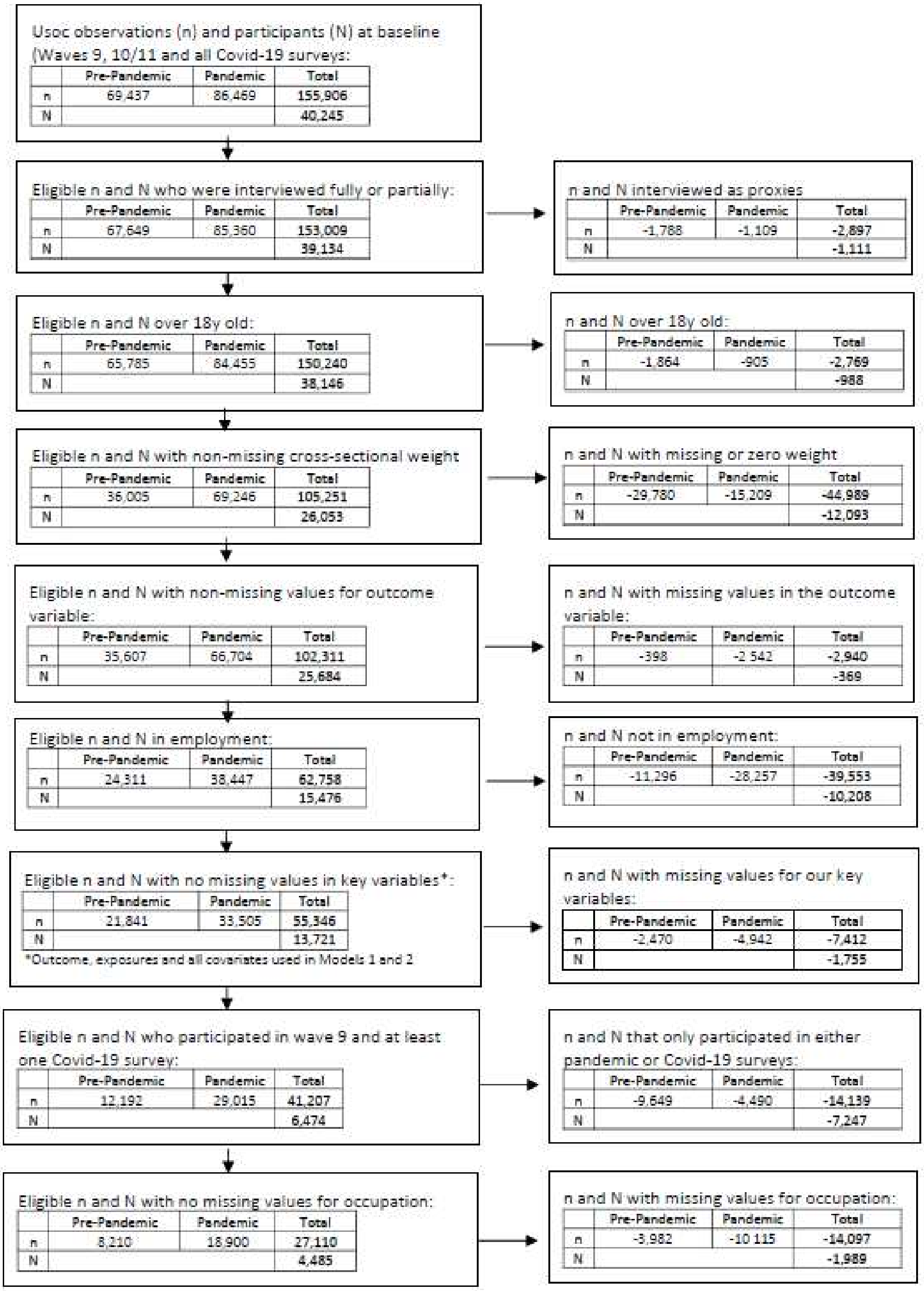

Figure S2. Stratified Regression models (UK Country of residence)

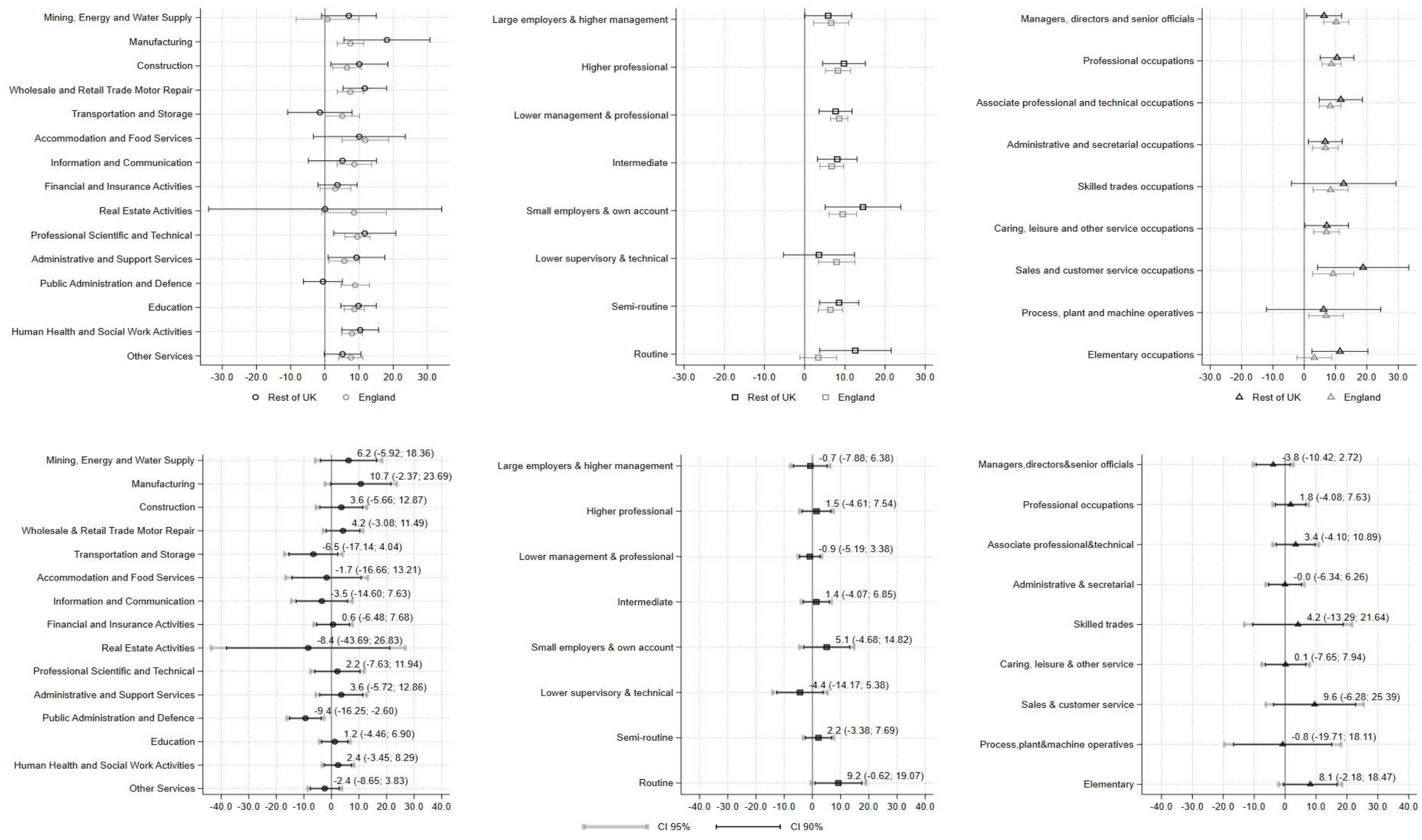

Note: circles represent industries, squares social class and triangles occupational groups

Figure S3. Stratified Regression models (Age)

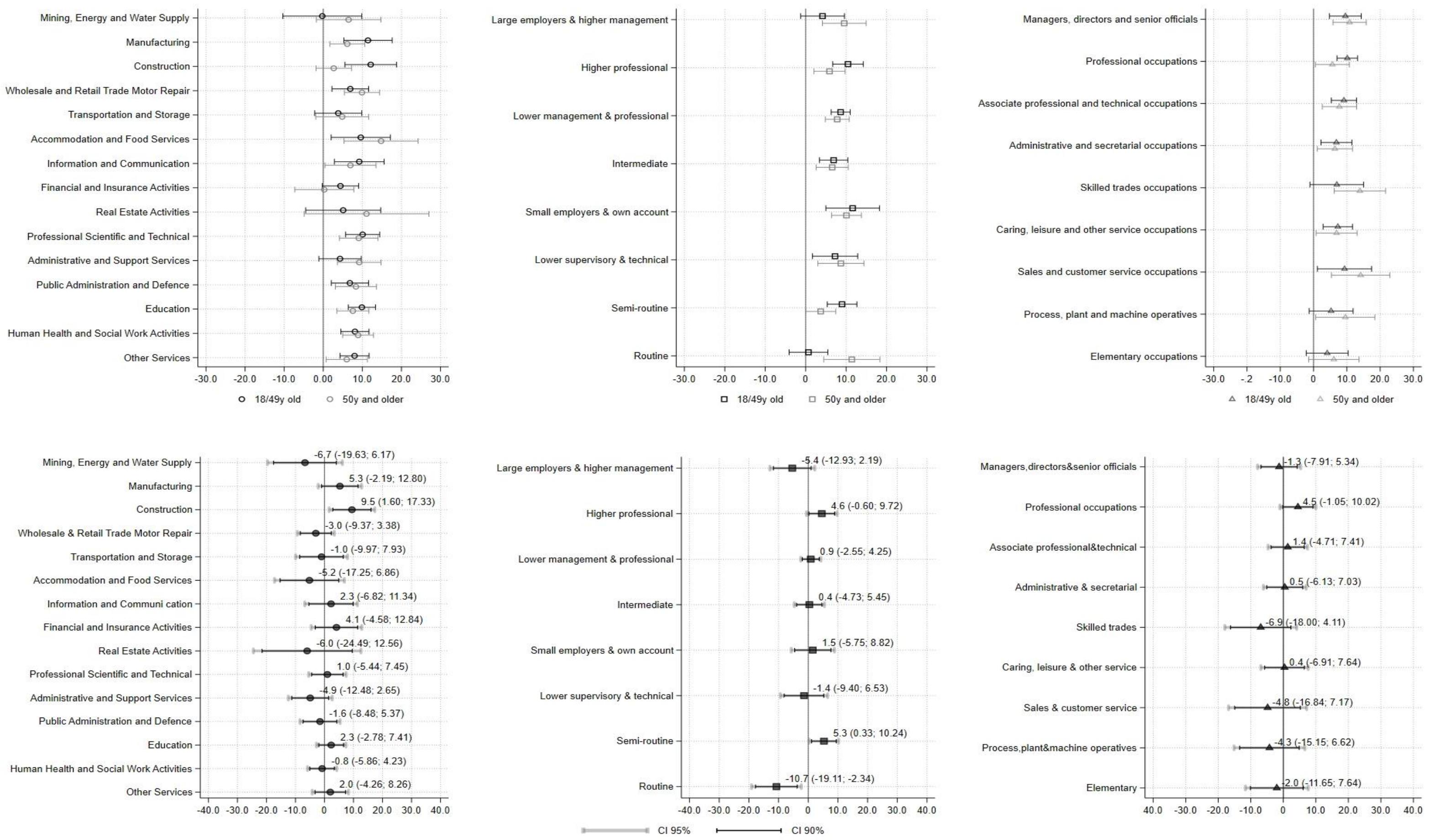

Note: circles represent industries, squares social class and triangles occupational groups

96 Figure S4. Distribution of observations in our analytical sample across all different values of GHQ-12.

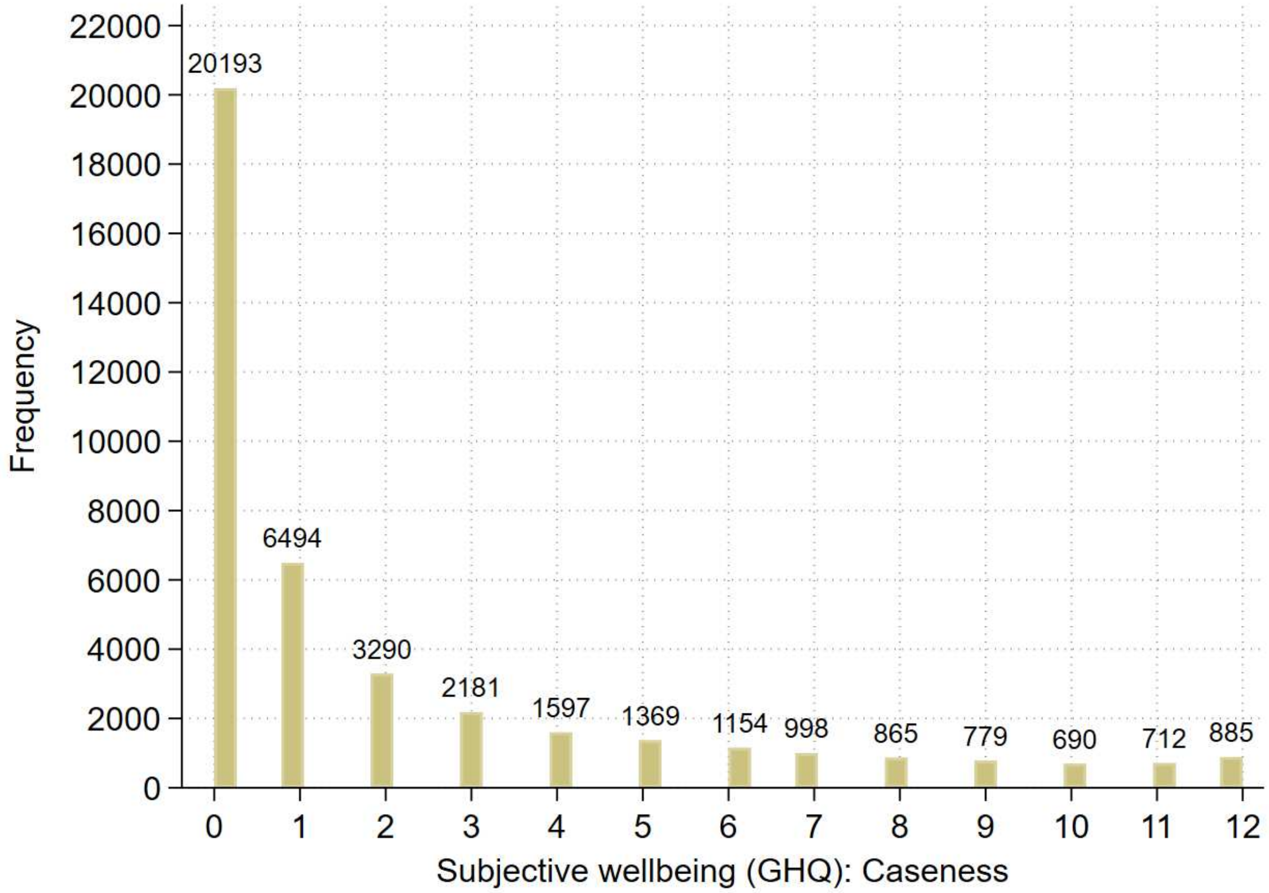

97
